# User Actions within a Clinical Decision Support Alert for the Management of Hypertension in Chronic Kidney Disease

**DOI:** 10.1055/a-2554-3969

**Published:** 2025-07-02

**Authors:** Lipika Samal, Sarah W. Chen, Stuart Lipsitz, Heather J. Baer, John L. Kilgallon, Michael Gannon, Ryan Dunk, Weng Ian Chay, Richard Fay, Michael Sainlaire, Chenxi Gao, Matthew Wien, Pamela M. Garabedian, Edward Wu, Hojjat Salmasian, David W. Bates, Patricia C. Dykes, Adam Wright, Allison B. McCoy

**Affiliations:** 1 Division of General Internal Medicine, Brigham and Women's Hospital, Boston, Massachusetts, United States; 2 Harvard Medical School, Boston, Massachusetts, United States; 3 Division of Biostatistics, Harvard T.H. Chan School of Public Health, Boston, Massachusetts, United States; 4 Division of Epidemiology, Harvard T.H. Chan School of Public Health, Boston, Massachusetts, United States; 5 Hackensack Meridian School of Medicine, Nutley, New Jersey, United States; 6 Eastern Virginia Medical School, Norfolk, Virginia, United States; 7 Department of Clinical and Quality Analysis, Mass General Brigham, Somerville, Massachusetts, United States; 8 Alabama College of Osteopathic Medicine, Dothan, Alabama, United States; 9 Children's Hospital of Philadelphia, Philadelphia, Pennsylvania, United States; 10 Department of Biomedical Informatics, Vanderbilt University, Nashville, Tennessee, United States

**Keywords:** clinical decision support, alert, override reasons, user actions, feedback

## Abstract

**Objective:**

This study aimed to examine user actions within a clinical decision support (CDS) alert addressing hypertension (HTN) in chronic kidney disease (CKD).

**Methods:**

A pragmatic randomized controlled trial of a CDS alert for primary care patients with CKD and uncontrolled blood pressure included prechecked default orders for medication initiation or titration, basic metabolic panel (BMP), and nephrology electronic consult (e-consult). We examined each type of action and calculated percentages of placed and signed orders for subgroups of firings.

**Results:**

There were firings for medication initiation (813) and medication titration (430), and every firing also included orders for nephrology e-consult (1,243) and BMP (1,243). High rates of override (59.6%) and deferral (14.6%) were observed, and CDS-recommended orders were only signed about one-third of the time from within the alert. The percentage of orders that were signed after being placed within the alert was higher for medication initiation than for medication titration (33 vs. 12.0% for angiotensin-converting enzyme inhibitors [ACEi] and 38.8 vs. 14% for angiotensin II receptor blockers [ARBs]). Findings suggest that users are hesitant to commit to immediate action within the alert.

**Conclusion:**

Evaluating user interaction within alerts reveals nuances in physician preferences and workflow that should inform CDS alert design. This study is registered with the Clinicaltrials.gov Trial Registration (identifier: NCT03679247).

## Background and Significance

Clinical decision support (CDS) systems can provide guideline-based recommendations based on patient-specific data. 1 2 CDS systems improve quality of care and reduce administrative burden, which is key given the amount and increased complexity of information available in electronic health records (EHRs). 3 4 5 Primary care physicians (PCPs) often care for patients with multiple chronic conditions which leads to competing demands, such as simultaneous responsibilities over multiple initiatives, projects, and research studies, as well as time constraints in meeting with patients. 6 7 8 9 10 11 12 13 Information hazards in EHRs including conflicting information and information overload can also cause unintended harm. 14 15 16 17

Early-stage management of chronic kidney disease (CKD) by PCPs is key to reducing poor outcomes. 18 19 20 Prior studies of CDS for CKD management have been successful in reducing glomerular filtration rate (GFR) loss, 21 increasing rates of diagnosis, 22 increasing urine albumin screening, 22 23 24 25 26 and increasing referral to nephrologists. 26 However, CDS designed to improve blood pressure control has not been successful in many prior studies. 21 22 24 25 27 28 29 Our previously published pragmatic clinical trial of CDS for CKD was the first to show a statistically significant effect. 30

## Objective

This study supplements our previously published work 30 31 32 33 34 where we described the design of the alerts and reported on the clinical trial results. This study focuses on user actions within the alert for laboratory testing, medication orders, and nephrology electronic consult (e-consult), as well as behavior such as overriding or deferring alerts and discontinuing medications after ordering them. By analyzing these actions and the comments provided by the user when overriding or deferring alerts, we aim to determine user acceptance of this CDS tool and assess the user's perception of its usefulness.

## Methods

### Pragmatic Clinical Trial Study Design and Results

The methods and results of the pragmatic clinical trial have been previously published. 30 31 In brief, we implemented the CDS across a network of 15 primary care clinics, randomized at the level of the PCP, and evaluated the impact of the CDS at the patient level. Adult patients who had a visit with a PCP at one of the clinics during the 2 years preceding the study enrollment period were eligible. Those meeting the inclusion criteria for CKD and had two instances of uncontrolled hypertension (HTN) in the 2 years preceding the enrollment period and at the enrollment visit were automatically included. The previously completed pragmatic clinical trial consisted of a comparator group in the usual care arm. The PCPs in the usual care arm did not have the opportunity to view or interact with the Best Practice Advisories (BPAs), and thus, the BPAs fired “silently.”

The primary outcome of the clinical trial was the change in mean systolic blood pressure (SBP) between baseline and 180 days, compared between arms. The study was not blinded. The study was conducted from February 26, 2021, to February 25, 2022, with data collection continuing until October 25, 2022. The final results of the study showed that the intervention was associated with a statistically significant difference in the primary outcome, change in mean SBP, and more patients received an action aligned with the CDS recommendations in the intervention group than in the usual care group. 30 This study was approved by the Mass General Brigham Institutional Review Board and the requirement for written consent was waived.

### Design and Functionality of the Clinical Decision Support Alerts

**Fig. 1:**
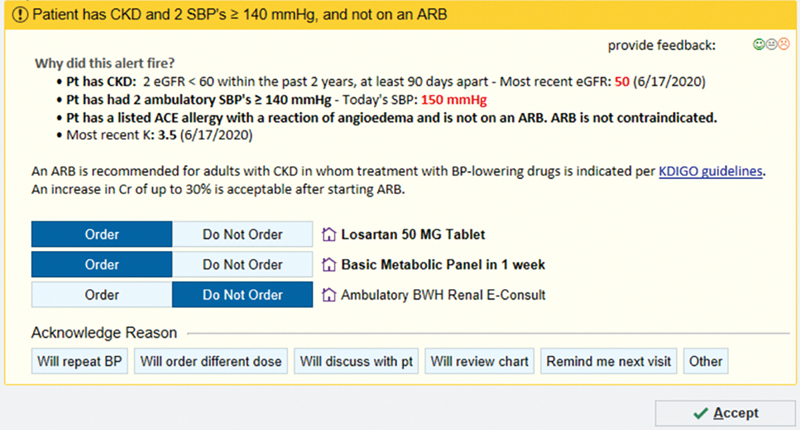
CDS alert (computable phenotype 1B) with explanation of trigger criteria, relevant clinical information, clinical practice guidelines, recommended actions, and required accountable justification (“Acknowledge Reason”; data presented are imaginary). (Reproduced with permission from Samal et al. 34 ) ACE, angiotensin-converting enzyme; ARB, angiotensin II receptor blocker; BP, blood pressure; CDS, clinical decision support; CKD, chronic kidney disease.

The CDS intervention was composed of a set of five BPAs implemented in Epic (Epic Systems, Verona, WI), the EHR system used at the study sites. The inclusion of patients for this pragmatic clinical trial was based on real-time assessment of two inclusion criteria: (1) CKD, defined by laboratory values, and (2) uncontrolled HTN, defined as at least one ambulatory SBP >140 mm Hg within the 2 years preceding the visit where the patient was assessed for inclusion in the study, as well as at that visit. The inclusion criteria were employed as computable phenotypes to estimate the frequency of alert firing prior to the clinical trial. 34 This data analytic phase of the project also allowed us to determine data adequacy for all of the data elements included in the logic. 35 BPAs used in this study were displayed as an interruptive alert to PCPs. Each BPA included display text (i.e., an explanation of trigger criteria, relevant clinical information, links to clinical practice guidelines), a prechecked default order for a medication, a prechecked default order for a basic metabolic panel (BMP), and an optional order for a nephrology e-consult ( Fig. 1 ). The optional order for a nephrology e-consult was added based on primary care leadership feedback and capacity for the nephrology service to fulfill requests was confirmed by our nephrology coinvestigator (G.M.M.). The specific dose and type of medication recommended by the BPA varied based on the patient's baseline regimen. For example, the alert that was fired for those who did not have an ACE/angiotensin II receptor blocker (ARB) on the medication list recommended the initiation of ACE lisinopril (5 mg) whereas the alert that was fired for those who had a documented allergy to ACE and no ACE or ARB listed on their medication list recommended initiation of ARB losartan (50 mg). 30 34 The alerts were a hard stop, meaning that the user was required to accept the alert, defer the alert, or provide a reason for overriding the alert. An iterative human-centered design process informed the design of the alerts. 32 The triggering action for the alerts was “Open Chart.” Other potential triggering actions were considered during the human-centered design phase of the project, but “Open Chart” was the only consistent time point across PCP workflows that would allow the PCP to prescribe the medication.

### User Actions within the Clinical Decision Support Alert

**Fig. 2:**
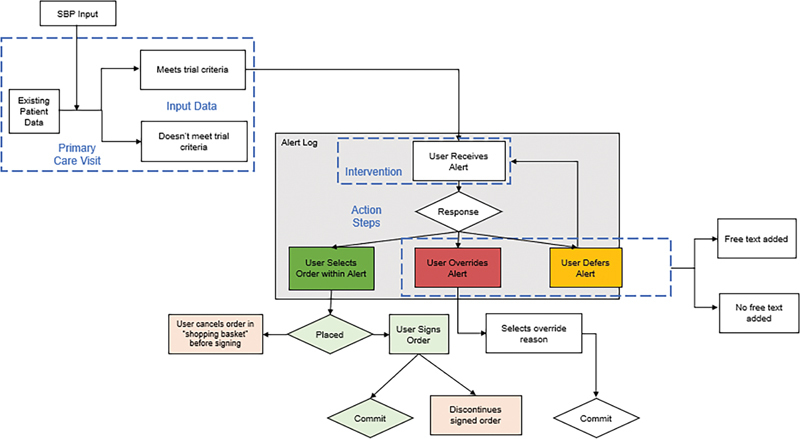
Alert workflow. SBP, systolic blood pressure.

Potential user actions are displayed in Fig. 2 . If the user did not want to submit the prechecked default order, he or she would click “do not order.” To close the alert, the user would be required to override or defer the alert. When overriding the alert, the user was required to enter an accountable justification from a list (“will repeat the blood pressure measurement,” “will order different dose,” “will discuss with patient,” “will review chart,” or “other,” with or without a free-text comment). When deferring an alert, the user was required to select the “remind me next visit” option from the response list. The percentage of alerts that were overridden or deferred indicates the number of instances in which the alert was deferred or overridden out of all instances where the alert fired.

If the user instead clicked “accept,” the prechecked default orders were placed in a shopping cart, where the user could choose to “pend,” “sign,” or “cancel” ( Supplementary Fig. S1 [available in the online version only]).

Data retrieval was completed by querying the data warehouse (Epic Clarity) for all actions completed within the alert during any instance in which the alert fired (intervention arm only). For example, a medication that was ordered from within the alert would be labeled “from BPA” whereas a medication that aligned with the alert recommendation but was ordered from outside the alert would be labeled as “same drug, route, and form.”

The percentage of orders that were placed within the alert indicates the number of instances in which an order was placed out of all firings in which the option to place that order was offered to the user by the alert. There were five alerts and each one fired a different number of times, depending on the patient's baseline medication regimen, so the denominator of firings was different for each alert. The percentage of orders that were signed indicates the number of instances in which an order was signed out of all instances in which the order was placed from the alert. After the order was signed, the order could be discontinued.

The percentage of orders that were discontinued indicates the number of instances in which an order was canceled after being signed, out of all the instances in which the order was signed from the alert. We examined the proportion of signed medication orders that were discontinued within 1 year after the trial period ended by categorizing the discontinuation reason entered by the PCPs. In cases where the discontinuation reason entered was “error,” we conducted a chart review including other medication orders, patient phone encounter documentation, and patient portal messages.

### Data Analysis

The data analysis for this study was conducted using data from patients in the intervention arm of the pragmatic clinical trial. We examined user actions such as orders for medications, laboratory testing, and nephrology electronic consults and determined which ones were placed and signed from the alert. We also examined user interaction with features to override or defer the alert. We categorized free-text comments according to the “Five Rights of CDS” (right information delivered to the right person, through the right intervention format and the right channel, and at the right time in workflow; Supplementary Table S1 [available in the online version only]). 36 Two members of the study team (L.S. and S.W.C.) independently reviewed and categorized the free-text comments.

## Results

### Clinical Characteristics and Alert Use

The intervention arm included 1,029 patients (65.1% female; 69.9% White, 19.6% Black, 11.8% Hispanic, and 2.5% Asian; mean ± SD age 74.2 ± 0.3 years; and 84.7% treated for HTN at baseline). 30 A total of 1,243 alerts were fired throughout the duration of the trial.

### User Action

#### Override

If a user chose to override the alert, they selected one reason from a list of response options. Out of all alert firings, 59.6% were alert overrides. The most commonly chosen override reasons were “will repeat the blood pressure measurement,” “will review chart,” and “will discuss with patient” ( Table 1 ).

**Table 1:** User action as indicated by a specific override or deferral reason selected within the alert

Override or deferral reason	*N* /Total firings (%)
Override	742/1,243 (59.6%)
Will repeat the blood pressure measurement	283/1,243 (22.7%)
Will order different dose	11/1,243 (0.9%)
Will discuss with patient	179/1,243 (14.4%)
Will review chart	188/1,243 (15.1%)
Other	81/1,243 (6.5%)
Deferral	
Remind me next visit	182/1,243 (14.6%)
Remind me next visit clicked greater than once	18/182 (10.0%)

Users entered 98 free-text comments ( Table 2 ; Supplementary Table S1 [available in the online version only]). Common themes included deferral of responsibility to another physician, the patient's inability to tolerate the recommended medication, disagreement with the alert recommendation, and disruption to PCP workflow.

**Table 2:** Representative free-text comments entered by users who overrode the alert, categorized according to the “Five Rights of CDS: right information delivered to the right person, through the right intervention format and the right channel, and at the right time in workflow”

Category	Representative free-text comments (verbatim) a
Right information	
Recommended medication was not tolerated previously	“angioedema to ACE!,” “aki,” “h/o hyperK,” “ho anaphylaxis”
On alternative medication	“On diltiazem for SVT instead,” “elevated K, will increase Toprol instead”
Disagreed with alert recommendation	“Repeat BP normal,” “pt is elderly and within range of SBP <160,” “white coat syndrome,” “home BP excellent,” “Does not have CKD,” “permissive HTN”
Will monitor or take further actions	“Pt will check BP at home and report,” “Starting 5 mg, h/o elevated K in past w/ ACEI so will start w/ 5 mg and monitor carefully”
Agreement, though alert was overridden	“already discussed,” “done,” “Taken off HCTZ, bmp today, already seen by Renal”
Right person	
Alert is not the recipient's responsibility	“Not PCP,” “labile BP, managed by specialists,” “not appropriate, seeing neph”
Right intervention format and right channel	
Other	“Pt refuses,” “x”
Right time	
Deferring due to other priorities	“going to the ED,” “on hold due to renal function,” “defer”
Other	“PLEASE ELIMINATE A BPA THAT STOPS ME FROM STARTING A VISIT,” “Never met him yet. This field is too early”

Abbreviations: ACE, angiotensin-converting enzyme; ACEi, angiotensin-converting enzyme inhibitor; ARB, angiotensin II receptor blocker; BP, blood pressure; BPA, Best Practice Advisory; CDS, clinical decision support; CKD, chronic kidney disease; HCTZ, hydrochlorothiazide; HTN, hypertension; PCP, primary care physician; SBP, systolic blood pressure.

a Representative comments for each category are shown here. A full list of free-text comments is in Supplementary Table S1 (available in the online version only).

#### Deferral

Out of all alert firings in the intervention arm, 14.6% ( *n* = 182) were deferred. In 10% of these cases ( *n* = 18), the PCP continued to defer the alert again at the next visit and, in some cases, repeatedly over several visits ( Table 1 ).

#### Medication, Laboratory Testing, and Nephrology Electronic Consult Orders Placed and Signed

**Fig. 3:**
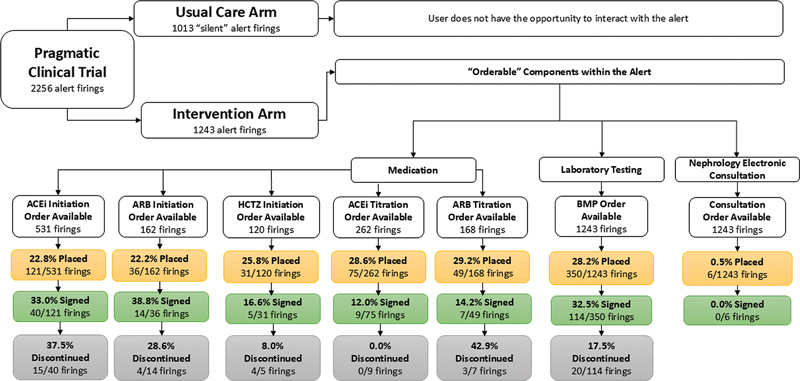
User action as indicated by medication, laboratory testing, and nephrology electronic consult orders placed and signed within the alert. Medication order recommendations were dependent on the participant's baseline antihypertensive medication regimen. ACEi, angiotensin-converting enzyme inhibitor; ARB, angiotensin II receptor blocker; BMP, basic metabolic panel; HCTZ, hydrochlorothiazide.

Orders that were placed within the alert were often not subsequently signed ( Fig. 3 ). The percentage of signed orders, out of those that were placed, for medication initiation was higher than the percentage of signed orders placed for medication titration (33% vs. 12.0% for angiotensin-converting enzyme inhibitors [ACEi] and 38.8% vs. 14% for ARBs). The percentage of signed orders, out of those that were placed, for initiation of hydrochlorothiazide (HCTZ) was 16.6%.

The percentage of orders for laboratory testing (BMP) placed, out of all firings in which the option was available, was 28.2%. Of the placed orders, the orders were only signed 32.5% of the time. No orders for a nephrology electronic consult were signed ( Fig. 3 ).

### Medication Discontinuation

The most common reasons for discontinuation of medication orders within a 1-year period included “re-order,” “no longer taking,” and “error.” A chart review of the “error” cases ( *N* = 8) revealed that there were four cases where the patient communicated the error to the PCP. For example, one patient message described, “I was looking at my visit summary and noticed there is a medication on my list that I have never taken and do not now. It's losartan 50 mg.” the PCP responded, “It looks that it may have been a misunderstanding and I ordered it at your last visit. I will discontinue it in your chart.”

## Discussion

More than half of alerts were overridden. Users most commonly selected “will repeat the blood pressure measurement,” “will review chart,” and “will discuss with patient,” as the reasons for overriding the alert, which suggests that the PCPs read and understood the recommendation, but had clinically appropriate reasons to delay the decision to prescribe the recommended medication. In alignment with these findings, when an order was placed using the prechecked default order, the order was only signed about one-third of the time. Again, this suggests that the PCP accepted the recommended prescription, but, in the majority of cases, changed their mind before signing the order. Of orders that were placed from the alert, orders for medication initiation were more commonly signed than orders for medication titration. Overall, the findings are consistent with other studies of user acceptance of CDS alerts.

A major issue with the design of the intervention was that the triggering action was “Open Chart.” Given that the alert was a hard stop, this was disruptive to workflow. Future CDS studies must explore other ways to deliver non-interruptive CDS alerts that are useful to clinicians. Also, in our study, it may have made sense to deliver separate alerts for medication recommendations and nephrology e-consult because the beginning of the visit was not an ideal time to recommend referral to nephrology. 37 One interesting finding was that users chose to defer alerts by clicking “remind me next visit” about one-sixth of the time and, in some cases, continued to defer the alert. This may indicate that the information was valuable but that competing demands, time constraints, and workflow disruption precluded immediate action; however, it also may be interpreted as a workaround, as PCPs were not required to provide accountable justification for this decision. It is also possible that the COVID-19 pandemic may have contributed to alert deferrals, as this trial was conducted from February 2021-February 2022 which is a period in which many patients were returning to in-person care following a period of telehealth or a pause in care.

The impact of CDS on clinical practice guideline adherence and health outcomes for patients with CKD has been tested in several studies, though few studies have found a meaningful impact on ACEi/ARB prescription rates or improving blood pressure control on a population level. 21 22 24 30 Our previously published trial was the only one to show a significant reduction in SBP as the primary outcome within a CKD population. 30 We attributed this to the incorporation of human-centered design and behavioral economic principles in the development of the CDS. The current study shows that, in a majority of cases, PCPs did not proceed in signing orders placed from within the alert. Yet, our previously reported results show that PCPs did place and sign orders aligned with the CDS recommendation more often in the intervention arm than in the usual care arm (49.9% vs. 34.6%). 30 This comparison implies that, while some PCPs may not have signed and placed orders directly through the CDS intervention, the suggestions may nevertheless have influenced their behavior in signing and placing orders outside of the CDS.

Barriers that we were unable to address in the alert design were disruption to PCP workflow, alert fatigue, and the inability of users to filter recommendations. 38 39 40 41 Our findings of alert override and deferral are consistent with existing literature about interruptive versus non-interruptive alerts. 42 However, our alert design incorporated the concept of accountable justification, in which PCPs were prompted to enter a reason for deferring or overriding the alert, which gives us insight into why they overrode the alert. Free-text comments for overrides indicated several reasons for overriding including that PCPs felt they were not primarily responsible for CKD management if a specialist was seeing the patient. Understanding alert override reasons can facilitate improvements to alert logic and identify CDS malfunctions. 43 44 45 46 Some studies have examined deferral reasons for CDS alerts addressing ophthalmology referrals and vaccine rates. 47 48 Our study is the first to report on deferral reasons for CDS related to CKD management.

A notable finding is that there were four cases where it appears that the user placed and signed a medication order in error. Prior research indicates that technology-induced errors are an unintended consequence of a hard-stop alert design. 49 50

### Study Limitations

This study has several limitations. This study used a cross-sectional design and reported data from the intervention arm of a prior study so there is no comparator group. A user was able to perform two actions at once: (1) place an order and (2) select a deferral or override reason. We were unable to determine how often the user performed both actions, so our results are reported at the action level, rather than at the encounter level, in effect “double counting” those cases. The setting is a network of primary care clinics affiliated with an academic medical center located in a metropolitan setting. Therefore, the user actions observed in this study may not be generalizable to other groups of PCPs in the United States or internationally. Finally, we did not perform a formal qualitative analysis of the user free-text comments, but instead attempted to categorize the comments according to an existing framework (“Five Rights of CDS”).

## Conclusion

Examining user actions within CDS alerts for HTN management in a CKD population revealed that most alerts were overridden, and the majority of orders placed from within the alert were never signed. Yet our previous study, which included a comparator arm, showed that orders that aligned with the recommendations were placed and signed more commonly in the intervention arm than in the usual care arm. Studies of user interaction with alert features and comparison to other actions taken within the same encounter may inform changes to alert design that will maximize usefulness.

## Clinical Relevance Statement

This study examines user actions within a CDS alert that provides evidence-based recommendations to address HTN management for patients with CKD. Evaluating primary care provider feedback and usage of alert functions postimplementation reveals nuances in user preferences and clinical workflow. This information can inform future changes to CDS alert design to better address time constraints and alert fatigue in primary care.

## Multiple-Choice Questions

1. What is the benefit of including override and deferral reasons within the CDS alert design?
  - Encourage clinicians to consider the alert content more carefully
  - Inform meaningful changes to make to the alert design
  - Identify CDS malfunctions
  - All of the above.
  **Correct Answer** : The correct answer is option d. The concept of accountable justification requires users to report their rationale which has been shown to improve decision-making accuracy. Free-text comments highlight barriers to incorporating the alert within the clinical workflow, such as inappropriate timing or firing for the incorrect user, which may inform alert design changes and help identify malfunctions.
2. Based on user feedback, which of the following is NOT a significant barrier to CDS alert utilization in this study?
  - Competing demands within a patient encounter
  - Role delegation for co-management of patients with multiple chronic conditions
  - Inadequate provider education
  - Disruption of clinical workflow
  **Correct Answer** : The correct answer is option c. Evaluation of free-text comments highlights concerns primary care providers have when deciding to override the alert, such as inappropriate timing of the alert (d), deferring the alert due to other priorities (a), and deferring CKD management to a specialist (b).
