# Supplementary material for: User Actions within a Clinical Decision Support Alert for the Management of Hypertension in Chronic Kidney Disease
Source: Appl Clin Inform. 2025 Jul 2;16(3):595–603. doi: 10.1055/a-2554-3969 (PMC12221691; doi:10.1055/a-2554-3969)
Supplement: Supplementary file 1 — Supplementary Material [file 10-1055-a-2554-3969-s202405ra0180.pdf]

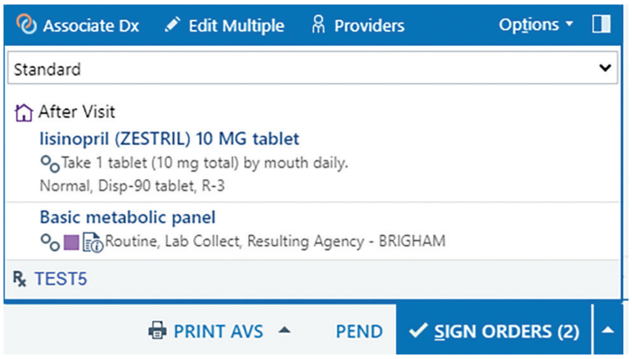

Supplementary Fig. S1 EPIC “shopping cart” feature which allows for PCPs to place an order from the BPA alert prior to signing. BPA, best practice advisory; PCP, primary care physician.

Supplementary Table S1 Free-text comments ($n = 98$) entered by users who overrode the alert, categorized according to the “Five Rights of CDS: right information delivered to the right person, through the right intervention format and the right channel, and at the right time in workflow”

Category	Free-text comments (verbatim)
Right information	
Recommended medication was not tolerated previously	Pt has a nephrologist and hasn’t tolerated meds before
	ace causes low sodium
	angioedema to ACE!
	did not tolerate
	did not tolerate HCTZ
	check allergies
	Cannot tolerate due to hyperkalemia and already has a nephrologist
	Angioedema to ACEi
	Already following with renal. Had AKI when attempted to trial in the past
	ace-i contraindicated
	Patient with hypercalcemia, on hctz
	ho anaphylaxis
	h/o hyperK
	Had AKI with ARB in the past. Will resume if BP log still high at home
	aki
On alternative medication	On diltiazem for SVT instead
	elevated K, will increase Toprol instead
	on losartan 100 mg
Disagreed with alert recommendation	repeat BP normal
	pt is elderly and within range of SBP <160
	white coat syndrome
	not applicable
	Pt has white coat
	BPs normal at home, white coat HTN
	home BP excellent
	n/a
	Does not have CKD
	INAPPROPRIATE POPUP

 © 2025. The Author(s).

Supplementary Table S1 (*Continued*)

Category	Free-text comments (verbatim)
	n/a
	Blood pressure well controlled
	Usually better controlled
	131/75
	permissive HTN
	Home BP normal
	Abnormally high for her
Will monitor or take precautions	in process
	i know
	Pt will check BP at home and report
	Starting 5 mg, h/o elevated K in past w/ ACEI so will start w/ 5 mg and monitor carefully
Agreement, though alert was overridden	already discussed
	Taken off HCTZ, bmp today, already seen by Renal
	done
	done
	ordered already
	followed by renal and BMP already ordered
	i already did this
	Just started ARB today
	will increase
	she just had BMP
Right person	
Alert is not the recipient's responsibility	already following with renal
	I am not PCP/
	Pt followed by Renal at BMC
	pt followed by renal
	called pcp
	followed by renal and transplant team
	Not PCP
	will discuss w pcp
	for PCP
	labile BP, managed by specialists
	already renal doc
	sees nephron
	followed by nephrology
	Re-referring back to renal (already established)
	has nephron
	not appropriate, seeing neph
	Followed by nephrology
	Followed by renal
	Already being seen by renal and chane in meds

(*Continued*)

 © 2025. The Author(s).

Supplementary Table S1 (*Continued*)

Category	Free-text comments (verbatim)
Right intervention format and the right channel	
Other	Stop this!
	Pt refuses
	other
	x
	NULL
	n
	o
	other
	other
	x
	other
	other
	x
	x
	I
	other
	x
	88
	Wh ocp/steroid tx rx?
Right time in workflow	
Deferring to other priorities	uc visit
	going to the ED
	on hold due to renal function
	TBD
	defer
	uc visit
	later
	pt has an appt
Other	alerting me before getting into my note is TOTALLY DISRUPTIVE and I can't think about this in this moment. HORRIBLE for workflow.
	PLEASE ELIMINATE A BPA THAT STOPS ME FROM STARTING A VISIT
	Never met him yet. This field is too early

Abbreviations: ACE, angiotensin-converting enzyme; ACEi, angiotensin-converting enzyme inhibitor; ARB, angiotensin receptor blocker; BP, blood pressure; BMP, basic metabolic panel; BPA, best practice advisory; CDS, clinical decision support; ED, emergency department; HCTZ, hydrochlorothiazide; PCP, primary care physician; SBP, systolic blood pressure; SVT, supraventricular tachycardia; TBD, to be determined.

 © 2025. The Author(s).
